# Short-term outcome after simultaneous pancreas-kidney transplantation with alemtuzumab vs. basiliximab induction: a single-center retrospective study

**DOI:** 10.1038/s41598-025-06750-y

**Published:** 2025-07-01

**Authors:** Tim D. A. Swaab, Robert A. Pol, Meindert J. Crop, Jan-Stephan F. Sanders, Stefan P. Berger, Hendrik S. Hofker, Stephan J. L. Bakker, Charlotte A. te Velde-Keyzer

**Affiliations:** 1 Department of Surgery, Division of Organ Donation and Transplantation, University Medical Center Groningen, University of Groningen, Groningen, The Netherlands; 2 Department of Internal Medicine, Division of Nephrology, University Medical Center Groningen, University of Groningen, Groningen, The Netherlands

**Keywords:** Pancreas transplantation, Immunosuppression, Induction, Alemtuzumab, Basiliximab, COVID-19, Outcomes research, Type 1 diabetes

## Abstract

During the COVID-19 pandemic our center adjusted the standard induction therapy for normal immunological risk simultaneous pancreas-kidney (SPK) transplantations from T-cell depletion by alemtuzumab (ALEM) to IL-2 receptor blocking by basiliximab (IL2R). Here, we analyze the impact of this change on 1 year post-transplantation outcomes. Thirty-six adult patients who underwent SPK transplantation between June 2015 and June 2023 were included, of whom 21 before February 2020 (ALEM) and 15 after February 2020 (IL2R). Patients were stratified into two groups based on the induction therapy received. One death occurred during the follow up period. A total of three pancreas and two kidney grafts were lost. No differences between kidney and pancreas graft function or rejection rates were observed. Patients receiving IL2R induction had significantly lower 30 day postoperative complication rates (34 vs. 46%, *p* = 0.03) and experienced fewer bacterial infections (< 6 months: 47 vs. 81%, *p* = 0.03). Additionally, lower rates of viral (including CMV) and fungal infections were observed. IL2R patients also had a significantly shorter hospital admission durations (14 vs. 30 days, *p* < 0.001). In conclusion, IL2R induction in SPK recipients was associated with similar short-term graft function and potentially improved outcomes compared to ALEM, warranting cautious interpretation due to sample size.

## Introduction

Type 1 diabetes (T1D) is characterized by beta cell destruction within the pancreas (usually immune mediated) and subsequent insulin deficiency^1^. Pancreas transplantation is considered the ‘gold standard’ of endocrine replacement therapy in T1D, providing physiologic glucose homeostasis through the restoration of endogenous insulin secretion^2^. Since the first successful pancreas transplant in December 1966, outcomes have markedly improved. This progress is attributed to advancements in surgical techniques, preservation methods, and most notably, immunosuppression^3^. As of 2023, it is estimated that over 67,000 pancreas transplants have been performed globally^4^.

Acute pancreas allograft rejection can occur after transplantation and has a significant negative impact on long-term graft function and survival^5^. The risk of acute rejection (cellular and/or antibody-mediated) is highest in the first six months^6^. To minimize this risk, immunosuppression induction therapy is initiated intra-operatively or shortly thereafter, followed by long-term oral maintenance therapy. Although the use of induction therapy in simultaneous pancreas kidney (SPK) transplantation is almost ubiquitous, there are many variations in dosage and choice of agents between different centers, and no universally accepted regimen exists. However, two commonly used induction regimens are T-cell depletion through means of alemtuzumab (ALEM) or anti-thymocyte globulin (ATG), and suppression of T-cell activation and proliferation through interleukin 2 receptor antagonism by basiliximab (IL2R)^7^. The immunosuppressive effects of IL2R are considered less potent and shorter-lived than those of ALEM^8^. While previous studies have investigated various induction regimens, recent and specific data comparing ALEM to IL2R induction in SPK transplantation remain scarce^9–12^.

The Coronavirus disease 2019 (COVID-19) pandemic significantly impacted solid organ transplantation around the globe. Initially, transplantation rates dropped, largely due to hospitals reallocating resources to manage COVID-19 patients. Later, the heightened risk for transplant recipients led many centers to restrict or suspend their transplant programs^13–15^. Our center opted to continue the pancreas transplant program with modification of the induction protocol for normal immunological risk SPK transplant recipients. For these patients our induction immunosuppressive therapy was switched from ALEM to less potent IL2R, accompanied by a slightly higher steroid dose. This alteration was motivated by the necessity to perform transplants with an acceptable risk of adverse outcomes in the case of COVID-19 infection while preventing further growth of the SPK transplant waiting list. In this study, we aim to assess the impact of this change in induction therapy on rejection rates, postoperative infections, surgical complications, and graft function within the first 365 days post transplantation.

## Methods

### Study design

This single center, retrospective study is part of a larger prospective research line investigating outcomes after pancreas transplantation. The study population consisted of adult patients who underwent a SPK transplant at the University Medical Center Groningen (UMCG) between June 2015 and June 2023. The indication for pancreas transplantation in all patients was T1D. To be eligible for inclusion patients needed to have a normal immunological risk. At our center, immunological risk is stratified based on donor-specific antibody (DSA) levels determined through Luminex single-antigen bead (LSA) testing. For this study normal immunological risk was defined as having no detectable DSA against human leukocyte antigen (HLA). Patient data were processed and digitally stored according to the Declaration of Helsinki ethical principles for research involving human subjects. The research line, of which this study is part, has been approved by the institutional review board of the UMCG (METc 2019/144). Due to the retrospective nature of this study, an exemption from the informed consent requirement was granted by the medical ethics committee of the UMCG. Additionally, our center’s objection registry for personal data use in medical research was consulted in accordance with our protocols. Patients who objected to the use of their data were excluded from the study. All research activities were conducted in line with the Declaration of Istanbul on Organ Trafficking and Transplant Tourism.

### Data collection

Donor characteristics were obtained from Eurotransplant donor reports. Baseline recipient data was retrospectively collected from the UMCG pancreas transplant database and supplemented where necessary using the patients’ digital medical records (Epic Systems, Wisconsin, U.S.A.). Collected data included donor and recipient age, sex, body mass index (BMI), type of dialysis prior to transplantation (hemodialysis (HD), peritoneal dialysis (PD), pre-emptive), dialysis vintage, duration of T1D, comorbidities, hospital admission duration, surgical complications, postoperative insulin or oral antidiabetic use, hospital readmissions and graft function parameters (estimated glomerular filtration rate (eGFR) and glycosylated hemoglobin (Hb1Ac)). Immunological data including HLA mismatches and panel reactive antibodies (PRA) were collected from immunology lab reports. Allocation of pancreas allografts within Eurotransplant is based on waiting time within the blood group strata and does not take the HLA (mis)match into account. The pre-existing comorbidities of the patients were evaluated using the Charlson Comorbidity Index (CCI)^16^. This index assigns scores to patients based on 19 predefined comorbidities, each weighted according to its severity. In this study, the CCI was employed to numerically quantify patients’ comorbidities, facilitating a more efficient and reliable statistical analysis. All infectious complications (excluding grade I) up until the time of hospital discharge were additionally scored in accordance with their Clavien Dindo grade and an adjusted (unvalidated) Comprehensive Complication Index specifically for infections was calculated (CDI-INF). All post-operative complications occurring within the first 30 days were graded based on severity through means of the Clavien-Dindo classification^17^. These were then subsequently used to calculate the Comprehensive Complication Index (CDI). The CDI is a quantitative measure that takes into account both the number and severity of complications. It is calculated using a formula that assigns weighted values to different complication grades^18^. Additionally, severe complications (greater than Clavien Dindo grade II) were recorded up until 180 days. Bacterial, viral and fungal infections were defined on clinical suspicion, culture and the initiation of empirical antibiotic, antiviral or antifungal therapy. Diabetes after transplantation was defined as return to oral antidiabetic medication and/or insulin (cases of pancreatic graft loss were also included). Delayed graft function was defined as the need for dialysis within the first week of transplantation^19^. Leucopenia was defined as a leukocyte count < 4.0 10^9^/L in accordance with our center’s protocol. Suspected cases of pancreas graft rejection were identified through clinical assessment utilizing imaging and serum pancreas enzyme levels. The estimated Glomerular Filtration Rate (eGFR) was calculated in accordance with the Chronic Kidney Disease Epidemiology Collaboration (CKD-EPI) Creatinine equation 2021^20^. Pre-transplantation eGFR for patients on dialysis was not calculated and set at 5 ml/min/1.73 m^2^.

### Surgical procedure

As a general principle, pancreas grafts are transplanted in a ‘head down’ orientation at our center. All pancreas transplants utilized enteric drainage through means of a side-to-side duodeno-jejunostomy with the proximal jejunum. Patients received 5000 IU of heparin prior to the vascular anastomoses being performed. Arterial anastomoses were made between the common iliac artery, or the external iliac artery, and the iliac Y-graft from the donor. The venous anastomoses were made with either the distal vena cava, common iliac vein or external iliac vein, based on graft size or previous abdominal procedures. For the kidney grafts vascular anastomoses were performed on the contralateral external or common iliac artery and vein. Bladder anastomoses were performed using the Lich Gregoir technique. All patients received postoperative anticoagulation with nadroparine (2850I/U 2dd) and platelet inhibition (acetylsalicylic acid 100 mg/1dd) and were routinely admitted to the intensive care unit for postoperative monitoring.

### Immunosuppression regimen

The induction therapy for SPK transplant recipients consisted of ALEM (2015-Jun 2020) or IL2R (Jun 2020-present), methylprednisolone, tacrolimus and mycophenolate mofetil. An overview of the induction therapy each of the two groups received during the first week is presented in Table 1. Maintenance immunosuppression consisted of tacrolimus (through level: intravenous (directly postoperative): >20 ug/l, oral initial 10–15 ug/l, 3–6 weeks 8–12 ug/l, 6 weeks to 6 months 6–10 ug/l) and mycophenolate mofetil (first two weeks 1000 mg/d, hereafter 750 mg/d) for both patient groups. In our center we titrate tacrolimus dose dependent on serum tacrolimus levels from post operative day 3 ensuring all patients achieved therapeutic tacrolimus levels within 5 days postoperatively. Patients receiving ALEM induction received a 5 mg/d maintenance dose of prednisolone. Patients receiving IL2R induction were started on 20 mg/d prednisolone daily for the first two weeks post-transplantation with the dosage being periodically decreased to 5 mg/d at 6 months post-transplantation.

**Table 1:** Immunosuppression induction therapy regimens.

Induction	Alemtuzumab (before 01-06-2020 )	Basiliximab (after 01-06-2020 )
Day 0	Methylprednisolone 500 mg + Alemtuzumab 30 mg s.c.	Methylprednisolone 500 mg + Basiliximab 20 mg
Day 1	Methylprednisolone 100 mg	Methylprednisolone 100 mg
Day 2	Methylprednisolone 100 mg	Methylprednisolone 100 mg
Day 3	Methylprednisolone 100 mg	Methylprednisolone 100 mg
Day 4	Prednisolone 5 mg	Prednisolone 20 mg + Basiliximab 20 mg
Day 5	Prednisolone 5 mg	Prednisolone 20 mg
Day 6	Prednisolone 5 mg	Prednisolone 20 mg
Day 7	Prednisolone 5 mg	Prednisolone 20 mg

### Viral prophylaxis

Cytomegalovirus (CMV) prophylaxis was risk-stratified and consisted of valganciclovir, with dosage adjustments based on recipient kidney function. The duration of prophylaxis varied depending on the donor and recipient CMV status, as well as the type of induction therapy. In both ALEM and IL2R induction no CMV prophylaxis was given if both donor and recipient were CMV IgG negative. In all other cases valganciclovir was given for six months in the ALEM group. In the IL2R group prophylaxis was given for three months in CMV IgG positive recipients and for six months in CMV IgG negative recipients with a CMV IgG positive donor.

### Bacterial/fungal prophylaxis

All patients received bacterial and fungal prophylaxis consisting of intravenous metronidazole, cefuroxime and fluconazole for five days post-transplantation. Oral cotrimoxazole was utilized as long-term prophylaxis for six months to prevent Pneumocytis jiroveci pneumonia, with dosage adjustments based on recipient kidney function.

### Statistical analysis

Patients were stratified into two groups for analysis based on the induction regimen they received. Data distribution was assessed through visual inspection using Q-Q plots and Wilk Shapiro analyses. Baseline characteristics and outcomes are presented as mean ± standard deviations (SD) for normally distributed continuous variables, median and interquartile range for non-normally distributed continuous variables and frequency (%) for categorical variables. To compare baseline patient characteristics and outcomes between patients receiving ALEM induction and IL2R induction, unpaired t-tests were employed for normally distributed continuous variables, Mann Whitney U tests for non-normally distributed continuous variables and chi-square tests were used for categorical variables. A two-sided p-value of ≤ 0.05 was considered significant. To evaluate the effect of preemptive transplantation versus dialysis dependence at the time of transplant on postoperative complications, we conducted a Univariate Analysis of Variance (ANOVA). Additionally, to assess the influence of the CDI-INF on hospital admission duration, we performed a linear regression analysis. Statistical analysis was performed using the Statistical Package for Social Sciences (IBM SPSS Statistics, version 28.0, Armonk, NY, USA).

## Results

Thirty-six recipients were included in the study, with 21 in the ALEM group and 15 in the IL2R group. Baseline characteristics are presented in Table 2. Mean age of patients was 42.2 ± 9.7 years and 64% were male. More than half (53%) of the patients underwent pre-emptive transplantation, while 42% was dependent on HD and two patients (6%) were dependent on PD. Patients transplanted before June 2020 (ALEM group), had a slightly longer dialysis vintage compared to those transplanted after June 2020 (IL2R group), with 25 months vs. 20 months, respectively. The majority of patients received grafts from donation after cardiac death (DCD) donors (DCD 64% (*n* = 23) vs. DBD 36% (*n* = 13)). Donor creatinine was not significantly different between groups (*p* = 0.24). The groups were immunologically well-matched, with a similar degree of total HLA mismatches (*p* = 0.24) and similar rates two mismatches for HLA A, HLA B, and HLA DR between the ALEM and IL2R groups (see Table 2). Additionally, the panel reactive antibody (PRA) was 0% for all patients. Overall, no statistically significant differences were observed in the baseline characteristics between the two groups.

**Table 2:** Baseline characteristics.

Characteristics	Total *N* = 36	ALEM *N* = 21	IL2R *N* = 15	*P*-value
Donor characteristics				
Donor type				0.77
DCD, n (%)	13 (36)	8 (38)	5 (33)	
DBD, n (%)	23 (64)	13 (62)	10 (67)	
Donor sex				0.25
Male, n (%)	25 (69)	13 (62)	12 (80)	
Female, n (%)	11 (31)	8 (38)	3 (20)	
Donor age (years)	32 [19.0-39.8]	34 [22.5–39.5]	22 [18.0–41.0]	0.32
Donor BMI (kg/m^2^)	23.1 ± 3.3	23.4 ± 3.4	22.7 ± 3.2	0.53
Donor creatinine (µmol/L)	60.5 [50.3–79.0]	54.0 [43.0-81.5]	66.0 [55.0–71.0]	0.24
Recipient characteristics				
Recipient age (years)	42.2 ± 9.7	42.2 ± 8.0	42.2 ± 12.0	0.98
Recipient sex				0.68
Male, n (%)	23 (64)	14 (67)	9 (60)	
Female, n (%)	13 (36)	7 (33)	6 (40)	
Recipient BMI (kg/m^2^)	24.9 ± 3.8	24.5 ± 4.4	25.5 ± 2.8	0.43
Diabetes duration (years)	30.1 ± 10.5	30.7 ± 8.3	29.3 ± 13.0	0.70
Dialysis type				0.97
Pre-emptive, n (%)	19 (53)	11 (52)	8 (53)	
HD, n (%)	15 (42)	9 (43)	6 (40)	
PD, n (%)	2 (6)	1 (5)	1 (7)	
Dialysis vintage (months)	23.1 ± 13.9	25.3 ± 13.8	20.1 ± 14.4	0.48
Charlson comorbidity index (CCI)	4.7 ± 1.1	4.9 ± 1.3	4.4 ± 0.6	0.21
Immunological characteristics				
HLA mismatches (total)	4 [3.0–5.0]	4 [3.0–5.0]	4 [3.0–4.0]	0.24
HLA A 2 mismatches, n (%)	10 (28)	7 (33)	3 (20)	0.38
HLA B 2 mismatches, n (%)	21 (58)	13 (62)	8 (53)	0.43
HLA DR 2 mismatches, n (%)	14 (39)	10 (48)	4 (27)	0.18
PRA at transplantation (%)	0	0	0	NA

Data are presented as mean ± standard deviations for continuous variables with a normal distribution, median with interquartile range for non-normal distribution and numbers (n) with frequency (%) for categorical variables. Performed *p* tests were unpaired t tests for continuous normally distributed variables, Mann Whitney U tests for continuous non-normally distributed variables and Chi^2^ for categorical variables.

DCD, donation after cardiac death; DBD donation after brain death; BMI, body mass index; HD, hemodialysis; PD, peritoneal dialysis.

### Postoperative outcomes

During the one-year follow-up period, one patient in the ALEM group died at 10 months post-transplant due to sepsis secondary to a diabetic foot infection. At the time of death, both the kidney and pancreas grafts were functioning. During the first six months following transplantation, four rejection episodes were documented, all in the ALEM group. These included three cases of combined kidney-pancreas rejection, occurring at 119, 167, and 180 days post-transplant, and one case of solitary pancreas rejection at 18 days post-transplant. Between six and twelve months post-transplant two additional rejection episodes were identified in the ALEM group, both involving kidney rejection, occurring at 204 and 264 days post-transplant. Diagnoses were based on clinical presentation, computed tomography, and biochemistry. Biopsy with subsequent histological confirmation of rejection was performed in four cases. Standard rejection treatment consisted of 1000 mg methylprednisolone for 3 days, however one patient was treated with a single 30 mg dose of ALEM. One of the patients with rejection concurrently developed a pancreatic fistula that did not respond to treatment, requiring a transplantectomy of the pancreas graft after 167 days. Another pancreas graft in the ALEM group and one kidney graft in the IL2R group were lost soon after transplantation due to severe graft thrombosis (2 days post-transplant) and primary non-function (4 days post-transplant), respectively. In the period of 6–12 months post-transplant one pancreas and one kidney graft were lost after 10 months due to death of the aforementioned patient. The incidence of delayed graft function (DGF) of the kidney differed between the ALEM and IL2R group (ALEM *n* = 9 [43%] vs. IL2R *n* = 2 [13%], *p* = 0.058).

**Fig. 1:**
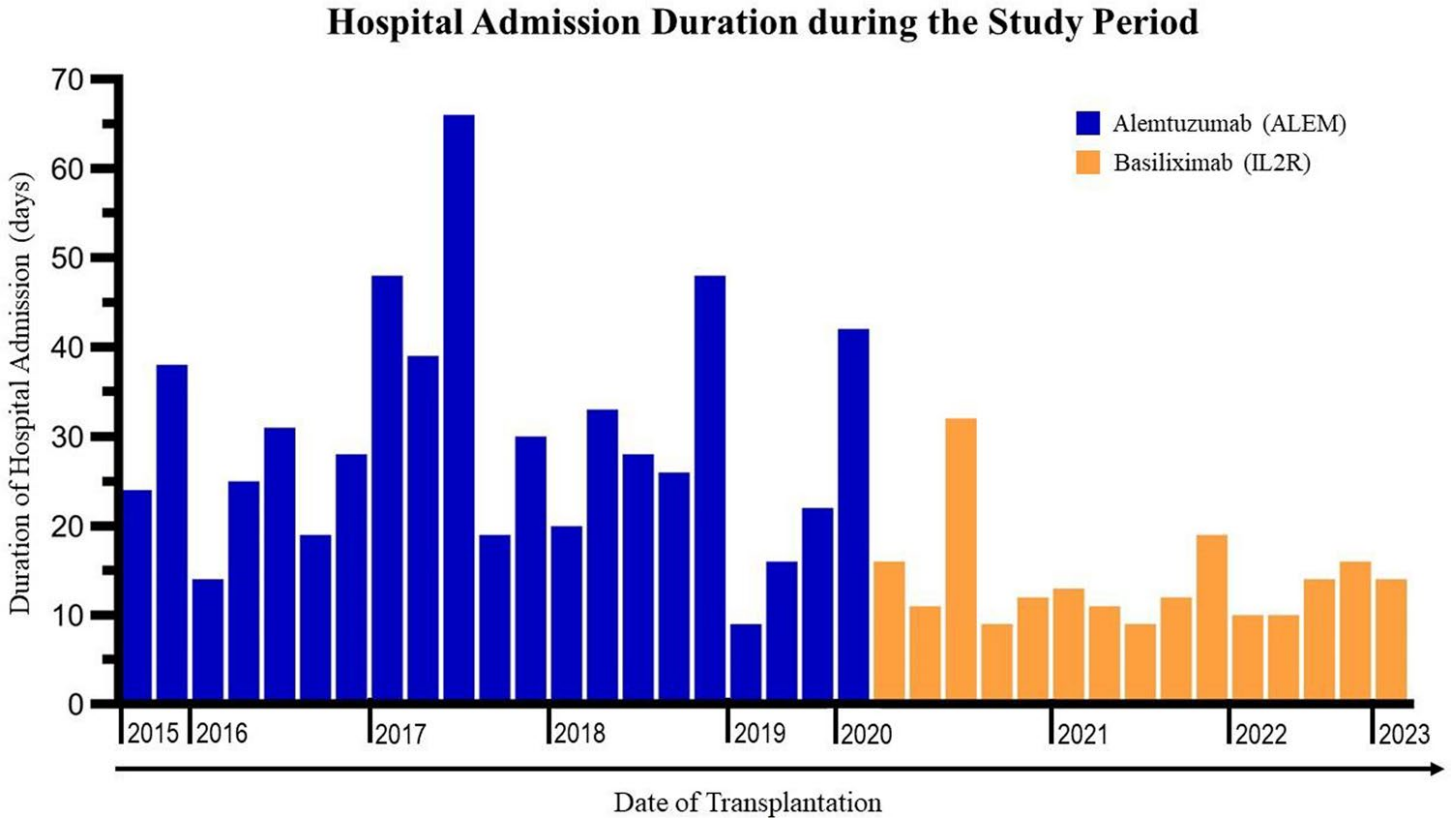
Chronological representation of patient admission duration.

A statistically significant difference was observed in the mean CDI within 30 days between the two groups (ALEM 46 ± 16 vs. IL2R 34 ± 15, *p* = 0.03). We additionally analyzed the influence of dialysis on postoperative complications until 30 days (CDI). Dialysis was not associated with postoperative complications in either group (ANOVA, ALEM *p* = 0.31 vs. IL2R, *p* = 0.56, data not shown). The CDI-INF was significantly lower in the IL2R group (ALEM 29.6 [20.6–34.9] vs. IL2R 0.0 [0.0–0.0], *p* = < 0.001). However, there was no significant difference in severe complications up to 180 days (*p* = 0.56) between the two groups. In the first 6 months post-transplant bacterial infections were significantly less frequent in the IL2R group (ALEM *n* = 17 [81%] vs. IL2R *n* = 7 [47%], *p* = 0.03). No significant difference was seen in the incidence of viral (*p* = 0.16) or fungal (*p* = 0.47) infections. In the period of 6 to 12 months post-transplant no statistically significant differences in bacterial (*p* = 0.39), viral (*p* = 0.72) or fungal infections (*p* = 0.07) were observed between the two groups. There were more transplants from a CMV IgG positive donor to a CMV IgG negative recipient in the IL2R group (ALEM *n* = 2 [9%] vs. IL2R *n* = 5 [33%]). One patient in the IL2R group experienced a CMV reactivation, while two patients in the ALEM group experienced a primary CMV infection post-transplantation. In the period 6 to 12 months post-transplant one patient in IL2R group experienced primary CMV infection. Overall, no statistically significant differences were observed in postoperative CMV infections between the two groups. An overview of the CMV serology can be found in supplementary Table 1. The overall primary hospital admission duration differed significantly between the two groups, with patients in the IL2R group having a mean admission duration of 14 ± 6 days and ALEM patients 30 ± 14 5.8 days (*p* = < 0.001, see Fig. 1). A subsequently performed linear regression analysis showed that the CDI-INF was strongly associated with admission duration (p = < 0.001). An overview of the postoperative outcomes up until 6 months can be found in Table 3 and between 6 and 12 months in Table 4.

**Fig. 2:**
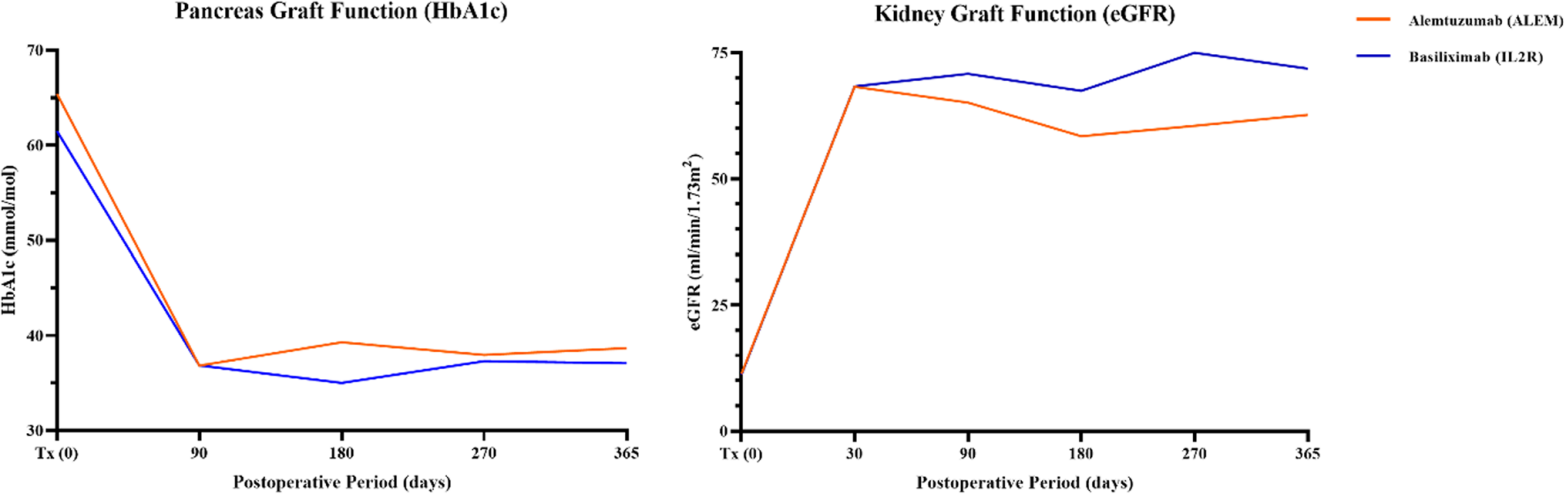
Postoperative pancreas and kidney graft function.

**Table 3:** Short term postoperative outcomes (until 6 months post-transplantation).

Characteristics	Total N = 36	ALEM N = 21	IL2R N = 15	*P*-value
Pancreas				
Pancreas acute rejection, n (%)	4 (11)	4 (19)	0 (0)	0.07
Graft thrombosis, n (%)	4 (11)	2 (10)	2 (13)	0.72
Diabetes after transplantation^a^, n (%)	5 (14)	4 (19)	1 (7)	0.29
Pancreatitis (all cause), n (%)	10 (28)	7 (33)	3 (20)	0.38
Graft loss, n (%)	2 (6)	2 (10)	0 (0)	0.22
Cold ischemia time (minutes)	514 ± 78	530 ± 76	494 ± 79	0.18
Warm ischemia time (minutes)	34 ± 9	36 ± 11	31 ± 5	0.10
Kidney				
Kidney acute rejection, n (%)	3 (6)	3 (14)	0 (0)	0.13
Delayed graft function, n (%)	11 (31)	9 (43)	2 (13)	0.06
Graft loss, n (%)	1 (3)	0 (0)	1 (7)	0.23
Cold ischemia time (minutes)	634 ± 167	655 ± 195	607 ± 123	0.41
Warm ischemia time (minutes)	40 ± 9	41 ± 10	37 ± 7	0.24
Surgical complications				
Severe complications^b^, n (%)	14 (39)	9 (43)	5 (33)	0.56
Infectious complications^c^ (CDI-INF)	10.3 [0.0–29.6]	29.6 [20.6–34.9]	IL2R 0.0 [0.0–0.0]	< 0.001
Comprehensive Complication Index (CDI)	40.9 ± 16.4	45.9 ± 16.2	34.0 ± 14.5	0.03
Leukopeniad, n (%)	16 (44)	11 (52)	5 (33)	0.26
Hospital readmission, n (%)	21 (58)	12 (57)	9 (60)	0.86
Reintervention, n of patients (%)	12 (33)	8 (38)	4 (27)	0.47
Rebleeding, n of events	5	2	3	
Drainage, n of events	4	3	1	
Transplantectomy, n of events	3	2	1	
Retransplantation, n of events	2	1	1	
Leakage^e^, n of events	2	2	0	
Other, n of events	7	7	0	
ICU admission duration (days)	1.6 ± 1.7	1.4 ± 1.0	1.9 ± 2.5	0.40
Total admission duration (days)	23.1 ± 13.4	29.8 ± 13.5	13.9 ± 5.8	< 0.001
Infections (< 6 months postoperatively)				
Bacterial infection, n of patients (%)	24 (67)	17 (81)	7 (47)	0.03
Urological, n of events	18	15	3	
Surgical, n of events	12	11	1	
Respiratory, n of events	3	1	2	
Gastrointestinal, n of events	2	1	1	
Soft tissue (non SSI), n of events	2	1	1	
Sepsis, n of events	1	1	0	
Other, n of events	5	3	2	
Viral infection, n of patients (%)	17 (47)	12 (57)	5 (33)	0.16
CMV, n of events	3	2	1	
Respiratory, n of events	6	3	3	
Gastrointestinal, n of events	3	3	0	
HSV-1 & 2, n	4	3	1	
Other, n	3	3	0	
Fungal infection, n (%)	4 (11)	3 (14)	1 (7)	0.47

Data are presented as mean ± standard deviations for continuous variables with a normal distribution, median with interquartile range for non-normal distribution and numbers (n) with frequency (%) for categorical variables. Performed *p*-tests were unpaired t-tests for continuous normally distributed variables, Mann Whitney U tests for continuous non-normally distributed variables and Chi^2^ for categorical variables.

ICU, intensive care unit; CMV, cytomegalovirus; SSI, surgical site infection.

^a^Diabetes was defined as return to exogenous insulin therapy or oral antidiabetic drugs (pancreas graft losses are also included).^b^Severe complications were defined as complications greater than Clavien Dindo grade II.^c^Infectious complication score up until moment of discharge.^d^Leucopenia defined as leucocyte count below 4.0 109/L. ^e^Enteric anastomotic leakage.

**Table 4:** Long term postoperative outcomes (6–12 months post-transplantation).

Characteristics	Total N = 36	ALEM N = 21	IL2R N = 15	P-value
Pancreas				
Pancreas acute rejection, n (%)	0 (0)	0 (0)	0 (0)	NA
Graft loss, n (%)	1 (3)	1 (5)	0 (0)	0.44
Kidney				
Kidney acute rejection, n (%)	2 (6)	2 (10)	0 (0)	0.22
Graft loss, n (%)	1 (3)	1 (5)	0 (0)	0.44
Infections (6–12 months postoperatively)				
Bacterial infection, n of patients (%)	15 (42)	10 (48)	5 (33)	0.39
Urological, n of events	7	4	3	
Surgical, n of events	2	1	1	
Respiratory, n of events	3	2	1	
Gastrointestinal, n of events	1	1	0	
Soft tissue (non-SSI), n of events	7	6	1	
Sepsis, n of events	1	1	0	
Viral infection, n of patients (%)	4 (11)	2 (10)	2 (13)	0.72
CMV, n of events	1	0	1	
Respiratory, n of events	2	1	1	
Herpes zoster, n of events	1	1	0	
Fungal infection, n (%)	4 (11)	4 (19)	0 (0)	0.07

Data are presented as mean ± standard deviations for continuous variables with a normal distribution and numbers (n) with frequency (%) for categorical variables. Performed *p* tests were unpaired t tests for continuous normally distributed variables and Chi^2^ for categorical variables.

ICU, intensive care unit; CMV, cytomegalovirus; SSI,  surgical site infection.

### Graft function

Throughout the course of follow-up, no significant differences were observed in kidney or pancreas graft function between the ALEM and IL2R groups. At 6 months, kidney function (eGFR) was similar (ALEM 58.5 ± 21.7 vs. IL2R 67.5 ± 24.5 ml/min/1.73 m², *p* = 0.26), as it was at 1 year (ALEM 62.7 ± 23.2 vs. IL2R 71.9 ± 25.5 ml/min/1.73 m², *p* = 0.29), as shown in Fig. 2. Pancreas function (HbA1c) at 6 months was also comparable (ALEM 39.3 ± 8.9 vs. IL2R 35.1 ± 3.6 mmol/mol, *p* = 0.13), as it was at 1 year (ALEM 38.7 ± 7.3 vs. IL2R 37.1 ± 3.7 mmol/mol, *p* = 0.46), as shown in Fig. 2. Within the first 6 months, three patients required antidiabetic medication (ALEM *n* = 2 [9.5%] vs. IL2R *n* = 1 [6.7%]). One patient in the ALEM group reverted to insulin treatment, attributed to insulin resistance (C-peptide > 1800 pmol/L) rather than pancreas graft dysfunction. The other two patients were managed with oral antidiabetic therapy (metformin). No new patients required insulin or oral antidiabetic therapy between 6 and 12 months.

## Discussion

This study identified a significant association between IL2R induction therapy and improved postoperative outcomes, specifically regarding the overall hospital admission duration, postoperative complications and bacterial infections relative to ALEM induction. Moreover, no significant differences were observed between the two groups in terms of rejection rates and graft function up until 1 year post-transplantation.

Acute pancreas allograft rejection is associated with a significant risk of graft failure and loss, explaining the widespread use of induction immunosuppression in pancreas transplantation^5,21^. Induction therapy aims minimize the risk of early rejection episodes and improve long term graft function and survival. Current induction therapies can be categorized into two main categories: T-cell depletion and T-cell activation and proliferation suppression. ALEM utilizes cytotoxic antibodies targeted against CD52 on T cell membranes, triggering an intracellular cascade resulting in apoptosis and T-cell depletion^22^. IL2R utilizes antibodies targeted at CD25 on T-cell IL-2 receptors inhibiting their function and suppressing T-cell activation and proliferation^23^. However, empirical evidence on the comparative risks and benefits of different induction therapies in pancreas transplantation remains scarce. None of the currently used induction agents are officially approved for use in pancreas transplant, with most evidence being extrapolated from clinical trials in kidney transplant recipients^24–31^. Performing large scale clinical trials in pancreas transplant recipients to address this issue remains challenging due to the relatively low number of pancreas transplants being performed per center^32^. Albeit various studies have investigated the impact of specific induction therapies on complications and patient/graft survival after SPK transplantation^9–12,33–40^. To our knowledge we are the fifth study specifically comparing ALEM to IL2R antagonists in the context of SPK transplantation. Three of these previously published studies utilize single-center retrospective cohorts at the University of Wisconsin^35–37^ (of which two cohorts partially overlap) and the fourth was a registry review (*n* = 8470), conducted by the University of Minnesota^40^. It is important to note that our population has more patients undergoing preemptive transplantation (ALEM 52% & IL2R 53%) than the populations utilized by Cerise et al. (ALEM 21% & IL2R 20%) and Pascual et al. (ALEM 39% & 28%). This higher prevalence of pre-emptive recipients in our study compared to prior series may reflect differences in center-specific selection criteria or allocation systems.

We observed no difference in both pancreas and kidney acute rejection rates within 1 year of follow-up. However, given the small cohort size, this study lacks the statistical power to draw definitive conclusions about rejection trends and these findings should therefore be interpreted with caution. A larger study which included SPK and solitary pancreas transplant recipients (*n* = 331) found no significant difference in pancreas allograft rejection rates (*p* = 0.12)^35^. In contrast, a smaller retrospective study by Pascual et al. (*n* = 136) at the University of Wisconsin reported significantly higher rates of acute cellular rejection in the kidney graft among patients receiving IL2R induction relative to ALEM^36^. Antibody-mediated rejection rates were similar between both groups. However, the leading cause of graft loss in the ALEM group was attributed to acute rejection whilst death with functioning graft was the predominant cause in the IL2R group. A recent study by Aziz et al., analyzing SPK and solitary pancreas transplant recipients at the University of Wisconsin between January 2011 and December 2019 (*n* = 471), identified that the acute pancreas rejection rate was significantly lower in the ALEM group (23%) compared to the IL2R group (34%, *p* = 0.02). However, a sub-analysis of the low immunological risk group found no significant difference in rejection rates (*p* = 0.08)^37^. We believe these findings underscore the importance of determining a patient’s immunological risk to allow for appropriate risk stratified induction therapy. At our center patients with normal immunological risk are presently allocated IL2R induction, whereas those with moderate risk are allocated ALEM induction. It’s noteworthy that the studies by Magliocca and Pascual^35,36^ did not stratify patients or perform sub-analyses according to normal or moderate increased immunological risk.

We observed no significant differences in pancreas and kidney graft function and survival between groups, in accordance with the studies conducted by Magliocca, Aziz and Cerise et al.^35,37,40^. In contrast, Pascual et al. observed increased Hb1Ac levels amongst patients receiving IL2R induction during the first 6 months of follow up (ALEM 5.1% ± 0.5% vs. IL2R 5.6% ± 0.4%, *p* = 0.02), which the authors attributed to the higher incidence of acute cellular rejection and subsequent steroid needs in IL2R-treated patients^36^. It’s important to note that our follow-up was limited to 1 year but all three studies performed by the University of Wisconsin suggest similar pancreas and kidney graft function and survival rates after periods of longer follow-up. Although differences in DGF rates were observed between the groups, they were not statistically significant, and we believe these differences are not attributable to the different induction therapies.

Within our cohort there were significantly less bacterial infections within the first 6 months post-transplant in the IL2R group (*p* = 0.03) and numerically lower rates of viral and fungal infections. These lower rates of bacterial, viral, and fungal infections were also observed during the six month to one-year follow-up period. Initially the reduced incidence of viral infections during the first six months could have been attributed to the effects of the valganciclovir CMV prophylaxis. Similar findings were reported by Aziz et al., who observed significantly lower rates of bacterial (ALEM 34% vs. IL2R 23%, *p* = 0.04) and CMV (ALEM 21% vs. IL2R 11%, *p* = 0.03) infections in patients receiving IL2R induction^37^. Magliocca et al. only analyzed viral infections (EBV, BKV and CMV) but also observed a statistically significant higher incidence of CMV infection in ALEM group (*p* = 0.002)^35^. Pascual et al. holistically reported opportunistic infections and observed a similar incidence of infection between groups (*p* = 0.613)^36^. It is important to highlight that the ALEM induction dosage utilized at the University of Wisconsin (2 × 30 mg) is higher compared to ours (1 × 30 mg), potentially impacting infection rates. Additionally, the viral prophylaxis differed between centers, with shorter CMV prophylaxis (only 90 days) given at the University of Wisconsin. The high degree of variability in infection reporting across the studies makes further extrapolation of the findings across studies difficult. While our results align with Aziz et al.‘s bacterial infection rates, these differences in reporting highlight the need for standardization in future studies to facilitate more direct comparisons amongst series.

To our knowledge this is first study to report a statistically significant decreased hospital admission duration in SPK transplant recipients receiving IL2R induction (ALEM 30 days vs. IL2R 14 days, *p* = < 0.0001).There was a statistically significant difference in the 30 day complication score (CDI) between the two groups (*p* = 0.03). An analysis of the influence of dialysis on postoperative complications showed no significant associations. The median CDI-INF varied significantly between IL-2R and ALEM groups, with patients in the IL-2R group exhibiting substantially lower scores (*p* = < 0.001). We subsequently performed a linear regression analysis between the CDI-INF and admission duration taking into account the induction therapy used. The overall model was highly statistically significant (*p* = < 0.001) and explained 61.8% (R^2^ = 0.618) of the variance in hospital admission duration. Thus the infection score was a significant predictor of hospital stay duration. Therefore we believe that the decreased hospital admission duration in the IL2R group was likely attributable to less postoperative complications and infections amongst IL2R patients, facilitating a more rapid recovery overall. Additionally, we graphed the hospital admission duration per-patient during the study period, which did not exhibit a temporal trend (Fig. 1). Nevertheless, given the sequential allocation of patients into ALEM and IL2R groups, it remains conceivable that a time-dependent variable associated with practice evolution may additionally influence the observed differences.

Our study does have some limitations that need to be addressed. The relatively small sample size and single-center design, combined with the highly ethnically homogenous population (Caucasian) in the Northern Netherlands, might limit extrapolation to other populations. We extensively report on postoperative infections however, the relatively short follow-up duration (1 year) although theoretically long enough to account for ‘rebound’ rejections following induction with T cell depleting agents^41,42^ hinders the assessment of long-term graft outcomes. Additionally, the retrospective observational study design hinders the establishment of causality regarding study outcomes. Future studies utilizing a multicenter, prospective approach with longer follow-up duration could mitigate these limitations. We are currently in the process of establishing a multicenter, prospective study with our partners in the European Pancreas Transplant Research Consortium (EPTRC).

In conclusion, this observational study demonstrated that IL2R induction for SPK transplants in patients with normal immunological risk was not inferior to ALEM induction, but even had more favorable postoperative outcomes Patients receiving IL2R induction displayed equivalent short-term graft function, lower rates of post-operative infections, and a shorter hospital admission duration compared to those receiving ALEM induction. Based on our findings, IL2R appears to be a safe and effective induction therapy for SPK recipients with normal immunological risk. Therefore, IL2R has remained the preferred induction regimen for normal immunological risk SPK transplants at our center since the COVID-19 pandemic.

## Data Availability

The dataset generated and analyzed during this study are not publicly available due to privacy of the patients but a pseudonymized version is available from the corresponding author upon reasonable request.
